# Carnosine selectively inhibits migration of IDH-wildtype glioblastoma cells in a co-culture model with fibroblasts

**DOI:** 10.1186/s12935-018-0611-2

**Published:** 2018-08-13

**Authors:** Henry Oppermann, Johannes Dietterle, Katharina Purcz, Markus Morawski, Christian Eisenlöffel, Wolf Müller, Jürgen Meixensberger, Frank Gaunitz

**Affiliations:** 1 Department of Neurosurgery, University Hospital Leipzig, Liebigstraße 20, 04103 Leipzig, Germany; 2 Medical Faculty, Paul-Flechsig-Institute of Brain Research, University of Leipzig, Leipzig, Germany; 3 Department of Neuropathology, University Hospital Leipzig, Leipzig, Germany

**Keywords:** Glioblastoma, Migration assay, Fibroblast ring co-culture, Carnosine

## Abstract

**Background:**

Glioblastoma (GBM) is a tumor of the central nervous system. After surgical removal and standard therapy, recurrence of tumors is observed within 6–9 months because of the high migratory behavior and the infiltrative growth of cells. Here, we investigated whether carnosine (β-alanine-l-histidine), which has an inhibitory effect on glioblastoma proliferation, may on the opposite promote invasion as proposed by the so-called “go-or-grow concept”.

**Methods:**

Cell viability of nine patient derived primary (isocitrate dehydrogenase wildtype; IDH1R132H non mutant) glioblastoma cell cultures and of eleven patient derived fibroblast cultures was determined by measuring ATP in cell lysates and dehydrogenase activity after incubation with 0, 50 or 75 mM carnosine for 48 h. Using the glioblastoma cell line T98G, patient derived glioblastoma cells and fibroblasts, a co-culture model was developed using 12 well plates and cloning rings, placing glioblastoma cells inside and fibroblasts outside the ring. After cultivation in the presence of carnosine, the number of colonies and the size of the tumor cell occupied area were determined.

**Results:**

In 48 h single cultures of fibroblasts and tumor cells, 50 and 75 mM carnosine reduced ATP in cell lysates and dehydrogenase activity when compared to the corresponding untreated control cells. Co-culture experiments revealed that after 4 week exposure to carnosine the number of T98G tumor cell colonies within the fibroblast layer and the area occupied by tumor cells was reduced with increasing concentrations of carnosine. Although primary cultured tumor cells did not form colonies in the absence of carnosine, they were eliminated from the co-culture by cell death and did not build colonies under the influence of carnosine, whereas fibroblasts survived and were healthy.

**Conclusions:**

Our results demonstrate that the anti-proliferative effect of carnosine is not accompanied by an induction of cell migration. Instead, the dipeptide is able to prevent colony formation and selectively eliminates tumor cells in a co-culture with fibroblasts.

**Electronic supplementary material:**

The online version of this article (10.1186/s12935-018-0611-2) contains supplementary material, which is available to authorized users.

## Background

Isocitrate dehydrogenase (IDH)-wildtype glioblastoma is the most malignant brain tumor of the adult brain and designated as Grade IV tumor by the World Health Organization (WHO) [1]. All tumors used in this study were IDH1R132H-non-mutant glioblastoma of elderly patients and, for reasons of simplicity, will further be referred to as GBM. Aside from a high mitotic activity and its ability to vascularize, GBM, as all diffuse glioma, has a high potential to infiltrate into intact brain tissue which makes it virtually impossible for the surgeon to completely remove the tumor. Cells able to migrate within intact tissue are considered to be the main cause of tumor recurrence which is generally observed within 6–9 month after surgery and standard therapy [2]. Therefore, any therapeutic approach has to consider that it may not be enough to inhibit the proliferation of cells, but should although prevent their spreading into intact tissue. Moreover, as Giese et al. [3] pointed out already more than 20 years ago, proliferation and migration appear to be mutually exclusive behaviors. The concept of a dichotomy of proliferation/migration has been observed by many groups and has coined the term “go or grow” [4]. Having this dichotomy in mind it is important that a substance that inhibits proliferation does not at the same time trigger migration and invasive behavior. This is the case for the dipeptide l-carnosine (β-alanyl-l-histidine). This naturally occurring dipeptide has been discovered in 1900 by Gulewitsch and Amiradzibi [5]. Aside from a number of physiological roles attributed to it, such as pH-buffering or the chelation of metal ions (for review see [6]), it is discussed as a potential drug for the treatment of tumors (for reviews see [7, 8]). After the first observations made by Nagai and Suda [9] and the rediscovery of its anti-neoplastic effect by Holliday and McFarland [10], carnosine’s anti-tumor effect has been shown in vitro for a variety of cells derived from different tumors. This, for instance, includes gastric cancer cells [11], colon cancer cells [12] and, with special emphasis to this work, cells derived from glioblastoma [13]. Unfortunately, the exact mechanisms by which the dipeptide exerts its anti-neoplastic effect are still unknown but appear to be pleiotropic and dependent on the tumor cells investigated (for review see [14]).

Although previous experiments pointed towards the possibility that carnosine also reduces migration and infiltration via inhibition of Matrix Metalloproteinase-9 in SK-Hep-1 hepatoma cells [15] and in oxygen–glucose deprived reactive rat astrocytes [16] tumor cell invasion in these experiments was determined using trans well chamber assays, which cannot answer the question whether migration into tissue or a layer of cells will also be inhibited by the dipeptide. The same is the case with recently published experiments performed with HCT-116 human colon cancer cells which also indicated that the invasion ability of these cells is significantly inhibited already at a concentration 0.5 mM carnosine [17]. Therefore, we analyzed the infiltrative capacity of IDH-wildtype glioblastoma cells in the presence of carnosine in a newly developed co-culture model. In our model glioblastoma cells were seeded inside a cloning ring placed in the well of a 12 well plate, seeding patient-derived fibroblasts outside the cloning rings. The rings were removed after the cells had attached to the culture dishes and the cells were incubated for different periods of time in the absence and presence of different concentrations of carnosine. Finally, the infiltrative potential of the tumor cells was analyzed by determining the number of colonies formed within the fibroblast layer and the area they covered.

## Materials and methods

### Reagents

Unless stated otherwise, all chemicals were purchased from Sigma Aldrich (Taufkirchen, Germany). Carnosine was kindly provided by Flamma (Flamma s.p.a. Chignolo d’Isola, Italy).

### Cell lines and primary cell cultures

The GBM cell line T98G, negative for IDH1R132H-mutation and *O*-6-methylguanine-DNA methyltransferase (MGMT) promoter methylation, was obtained from the ATCC, genotyped using a PowerPlex 21 System (Promega; Mannheim; Germany) by the Genolytic GmbH (Leipzig, Germany) and authenticated by comparison to data at the ATCC and the DSMZ. T98G cells were used at passage 5–7 after genotyping and authentication. Both, primary GBM cultures and primary fibroblast cultures were established from tissue samples obtained during standard surgery performed at the Neurosurgery Department of the University Hospital Leipzig during 2015 and 2016. All patients provided written informed consent according to the German laws as confirmed by the local committee. When possible, one primary GBM cell culture and one primary fibroblast culture was established from tissue samples obtained from each patient (Additional file 1: Table S1). All GBM samples were diagnosed and have been approved by the Neuropathology Department of the Leipzig University Hospital. IDH 1 status has been determined using immunohistochemistry and pyrosequencing, MGMT promoter methylation status was determined using nucleic acid amplification followed by pyrosequencing.

For cultivation, tissue specimens from the tumor, from galea or from periost were cut into approximately 1 mm^3^ large pieces and then separately placed into 25 mm^2^ culture flasks (TPP, Trasadingen, Switzerland) until tumor cells or fibroblasts grew out. When more than 90% confluence was reached specimens were removed and primary cell cultures were transferred into 75 mm^2^ culture flasks (TPP) for further cultivation. Cell cultures were maintained in high glucose DMEM (4.5 g glucose/ml) supplemented with 2 mM Glutamax™, 1% penicillin/streptomycin (all from Gibco Life Technologies, now Thermo Fisher Scientific, Darmstadt, Germany) and 10% FBS (Biochrom GmbH, Berlin, Germany), further referred to as “standard medium”, and kept in incubators (37 °C, 5% CO_2_/95% air).

### Cell viability assays

For cell viability assays, cells were counted and seeded into sterile 96-well plates (µClear, Greiner Bio One, Frickenhausen, Germany) at a density of 5000 cells/well in 200 µl standard medium. After 24 h of cultivation (37 °C, 5% CO_2_/95% air) the medium was aspirated and fresh medium supplemented with or without carnosine was added (100 µl/well) and the cells were incubated for additional 48 h. Then, the CellTiter-Glo Luminescent Cell Viability Assay (CTG, Promega, Mannheim, Germany) was employed to determine viable cells by measuring ATP in cell lysates and the CellTiter-Blue Cell Viability Assay (CTB, Promega) was used to quantify the cell’s metabolic capacity in living cells. All assays were carried out according to manufacturer’s protocols. Luminescence and fluorescence were measured using a SpectraMax M5 multilabel reader (Molecular Devices, Biberach, Germany).

### Co-cultivation of GBM cells and fibroblasts (ring-cultures)

When cells reached more than 90% confluence in 75 cm^2^ cell culture flasks they were detached using accutase (Thermo Fisher), counted and diluted for co-cultivation. The ring-cultures were established in 12-well plates. Therefore, sterile cloning rings (steel, 6 mm inner; 8 mm outer diameter, Hartenstein, Würzburg, Germany) usually used for the isolation of clones, were placed in the middle of each well dividing it into an inner-ring and an outer-ring part. Then, 2500 tumor cells suspended in standard medium (112 µl) were seeded inside the ring. Afterwards, 50,000 fibroblasts (in 658 µl standard medium) were seeded outside of the ring (Additional file 2: Figure S1). Co-cultures with cloning rings were incubated for 4 h (37 °C, 5% CO_2_/95% air) before the rings were carefully removed using sterile forceps. Medium was exchanged immediately after ring removal containing various concentrations of carnosine. On the following days medium was exchanged twice a week.

### Carnosine co-culture experiments

Carnosine was diluted in 0.7% NaCl solution and carnosine experiments were performed with concentrations of 0 mM, 10 mM, 25 mM, 50 mM and 75 mM. All ring-culture experiments were prepared as described above. Control experiments with T98G cells inside the ring and without fibroblasts in the outer part were kept for over 2 weeks. Ring co-cultures with T98G and fibroblasts (P0385) and with GBM cells and fibroblasts of the same patient (P0383 with P0385 and P0431 with P0433) were cultivated for 4 weeks. Throughout cultivation, cell growth and dissemination were monitored by bright field microscopy. After 4 weeks all co-cultures were fixated in 4.5% paraformaldehyde and stored in 1% sodium azide solution at 4 °C until microscopic analysis.

### Immunostaining

Immunofluorescent staining was carried out to discriminate between tumor cells and fibroblasts using anti-fibroblast TE-7 (CBL271, Merck Millipore, Darmstadt, Germany), anti-nestin (AB5922, Merck Millipore) and secondary antibodies (ab6563, ab150081, abcam, Cambridge, UK). Briefly, for the detection of TE-7 the fixated co-cultures were permeabilized with 0.1% TritonX-100 at room temperature (RT) for 5 min. After blocking with 10% goat serum for 15 min, samples were incubated with anti-fibroblast TE-7 primary antibodies (dilution 1:100) at 5 °C overnight, washed with TBS (20 mM Tris, 134 mM NaCl) and subsequently incubated with the secondary antibody (1:250; ab6563) for 45 min at RT. For the detection of nestin, fixated cell cultures were permeabilized with 0.1% TritonX-100 in TBS for 1 h at RT, blocked for 15 min with 10% goat serum and then incubated with an anti-nestin antibody (dilution 1:250) for 1 h at RT. Afterwards, cultures were washed with TBS and incubated with a dilution of secondary antibody (1:250; ab150081) for 45 min at RT. Finally, nuclei were counterstained with DAPI (4 µg/ml) and cell cultures were preserved in 10% sodium azide solution at 4 °C until microscopy.

### Microscopy

For microscopic analysis a Zeiss Axiovert 200M microscope (Zeiss, Oberkochen, Germany) equipped with a motorized stage (Märzhäuser, Wetzlar, Germany) with MosaiX software and by means of a CCD camera (Zeiss MRC) connected to an AxioVision 4.8.2 image analysis system (Zeiss) was used to create tile pictures. Each tile picture is composed of 285 single microscopic images taken at a magnification of 50 and represents a whole well of a 12-well plate. As denoted in the figure legend to Fig. 2 ImageJ images in this figure have been graphically enhanced for representation purposes using the Corel Draw Graphics Suite 2017 (Corel Corporation, Ottawa, Canada).

### Quantitative and statistical analysis

The number of colonies in ring co-culture experiments was determined using ImageJ after a color threshold and a common pixel size were defined. All pictures used for the analysis were taken at the same magnification and had the same size. Statistical analysis was performed using the algorithm for t-test implemented in Microsoft Excel 2010.

## Results

### Viability of patient derived fibroblasts and GBM cells under the influence of carnosine

**Fig. 1:**
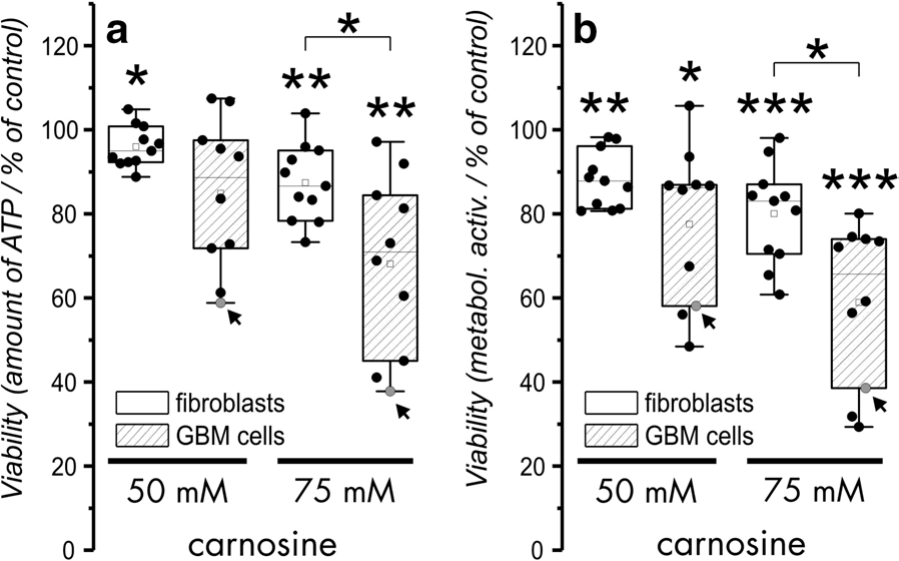
Viability of patient derived fibroblasts and primary glioblastoma cells under the influence of carnosine. Fibroblast cell cultures (11) and primary GBM cell cultures (9) were exposed for 48 h to different concentrations of carnosine (0, 50 or 75 mM). Viability was determined by measuring the amount of ATP in cell lysates (**a**) and by assessing metabolic activity (**b**). Each dot within the boxplots represents the mean obtained from six measurements of an individual cell culture. For comparison, the grey-colored dots (depicted by arrows) indicate the results obtained from experiments with the cell line T98G which was used for co-culture experiments. Statistical significance using data obtained from 11 fibroblast cultures and 9 primary GBM cultures was determined using Student’s t-test with: *p < 0.05; **p < 0.005; ***p < 0.0005

In order to figure out how fibroblasts isolated from patients who underwent surgery for GBM do respond to the presence of carnosine, 11 primary fibroblast cultures derived from galea or periost were exposed to different concentrations of carnosine. Viability was determined by measuring the amount of ATP in cell lysates and by the analysis of metabolic activity, as reflected by dehydrogenase activity. In order to compare the effect of the dipeptide on fibroblasts, we performed the same experiment with 9 primary GBM cell cultures derived from patients and cells from the GBM cell line T98G. All cells were seeded at a density of 5000 cells into the wells of 96-well plates and exposed to carnosine for 48 h. The result of the experiment is presented in Fig. 1. At a concentration of 50 mM carnosine, the amounts of ATP and the metabolic activity in fibroblasts were significantly reduced compared to untreated control cells (metabolic activity: 89.4% ± 7.53, p < 0.005; ATP: 96.6% ± 5.08%. p < 0.05). We also observed a significant reduction of metabolic activity in primary GBM cells treated with 50 mM carnosine (compared to untreated control: 79.7% ± 18.51%, p < 0.05). However, although the amounts of ATP were decreased at 50 mM carnosine (87.8% ± 16.35% compared to the untreated control) this effect was not significant (p = 0.056). Increasing the concentration of carnosine to 75 mM leads to a significant reduction of metabolic activity and a decrease in the amounts of ATP in both, primary GBM cells (metabolic activity: 61.2% ± 18.96, p < 0.0005; ATP: 71.5% ± 19.68%. p < 0.005) and fibroblasts (metabolic activity: 81.5% ± 12.03, p < 0.0005; ATP: 88.4% ± 5.07%. p < 0.005) compared to the untreated control. Furthermore, in the presence of 75 mM carnosine, viability of primary GBM cells was significantly stronger reduced than in fibroblasts (metabolic activity: p < 0.05; ATP: p < 0.05).

Please also note, that for statistical analysis data obtained from experiments with the cell line T98G were excluded.

### Outgrowth of cells from a ring culture without co-cultivated fibroblasts

**Fig. 2:**
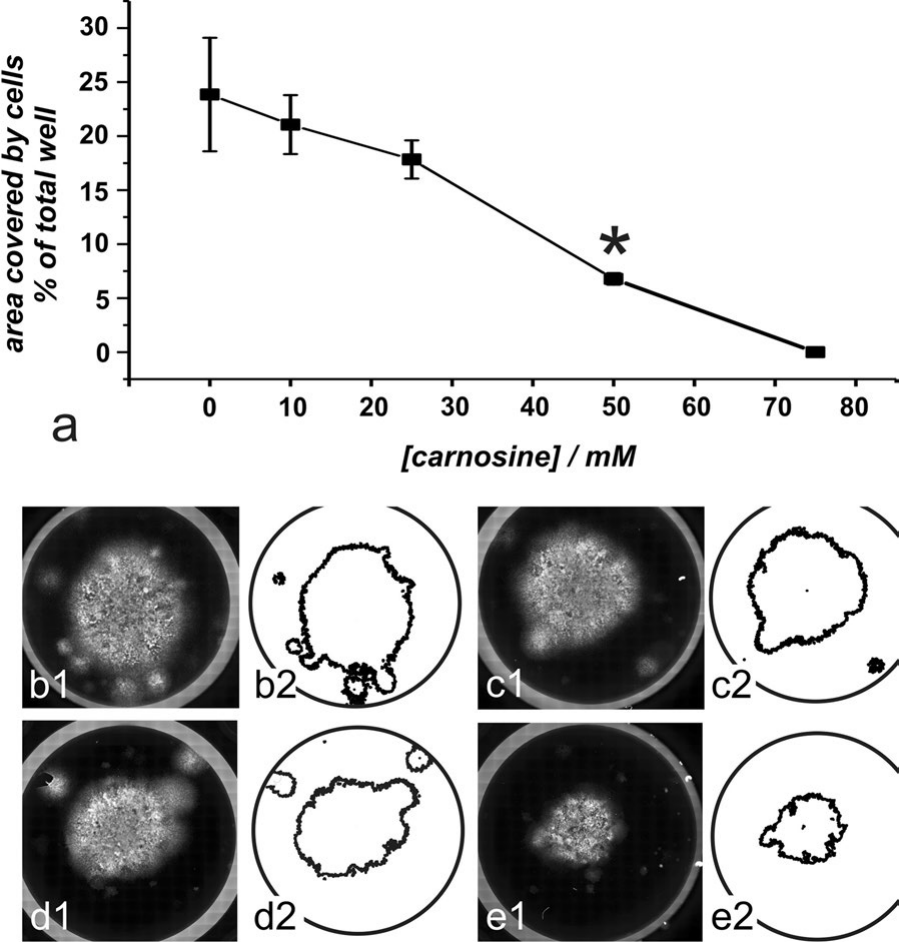
Outgrowth of T98G cells under the influence of carnosine. T98G cells were seeded inside a cloning ring placed in a 12-well plate. Rings were removed after 4 h and cells were allowed to grow in the presence of 0, 10, 25, 50 and 75 mM carnosine. After 14 days, images were taken and the area covered by tumor cells was determined. The mean and standard deviation of the covered area from four independent wells for each concentration are presented in **a**. A statistical significant reduction was only seen at 50 mM carnosine (*) and at a concentration of 75 mM carnosine no tumor cells were left on the plates. In the lower part of the panel examples of images are presented that were used for data analysis (**b** without carnosine; **c** 10 mM; **d** 25 mM and **e** 50 mM carnosine). #2 presents the corresponding ImageJ processed pictures (Note: for presentation purposes the pictures shown have been vectorized and image enhanced using Corel Draw Graphic Suite 2017)

Next, we asked how carnosine influences the growth of glioblastoma cells over a longer period of time when they start to grow after being seeded inside of a cloning ring and removal of it in the absence of other cells. Therefore, 2500 cells from the glioblastoma cell line T98G were seeded inside a cloning ring placed in a 12-well plate. Four hours later the rings were removed and the cells were allowed to grow for 2 weeks in the absence and presence of carnosine (10 mM, 25 mM, 50 mM and 75 mM). Medium was exchanged twice a week. After fixation of cells, 285 tiled images were taken from each well for each concentration employed (in quadruplicate) and the area covered by tumor cells was determined using ImageJ. The result of the experiment is presented in Fig. 2. As can be seen, the tumor cell covered area is continuously decreasing with increasing concentrations of carnosine although the effect becomes significant only at a concentration of 50 mM carnosine. At a concentration of 75 mM carnosine, no tumor cells were remaining on the plates, demonstrating that this concentration does not only inhibit proliferation but also eliminates the tumor cells when exposed to the dipeptide for 2 weeks. In conclusion, the experiment demonstrates that the cells do survive for 2 weeks when seeded at a density of 2500 cells inside a cloning ring and are incubated at carnosine concentrations of 50 mM or below.

### Fluorescent discrimination of tumor cells and fibroblasts in co-culture

**Fig. 3:**
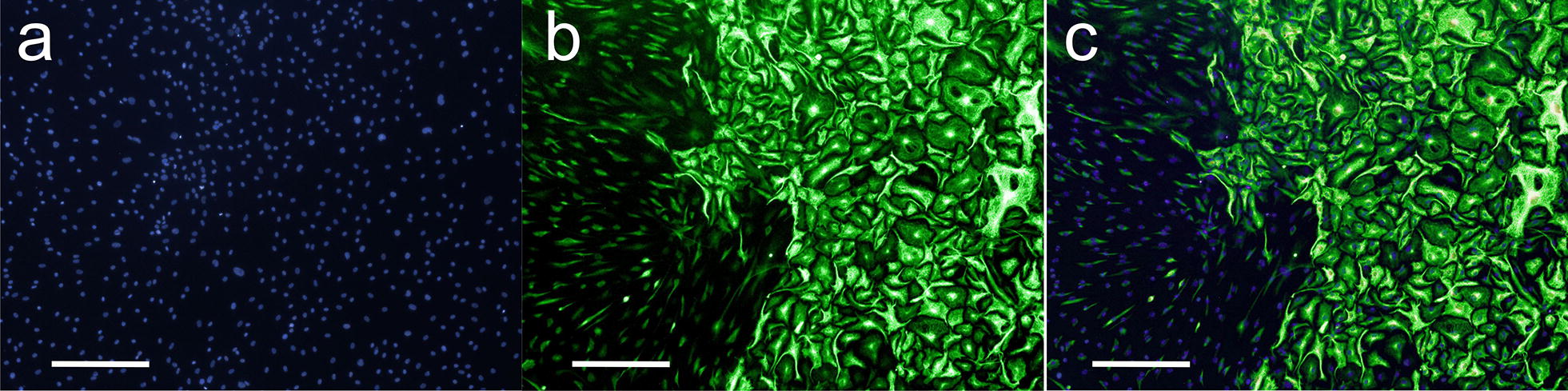
Nestin staining of patient derived GBM cells and fibroblasts in ring co-culture. The figure shows staining of a co-culture of fibroblasts (P0385) with GBM cells (P0383) with DAPI (**a**), a nestin-specific antibody (**b**) and the merged image (**c**). GBM cells are presented in bright green at the right side of the image. Scale bar: 300 µm

In order to perform co-culture experiments with GBM cells and fibroblasts, we had to establish a method by which both types of cells can be discriminated. Using patient derived fibroblasts (P0385) and primary glioblastoma cells (P0383) we were able to discriminate both types of cells using an antibody directed against nestin (Fig. 3). Although both types of cells were detected by the anti-nestin specific antibody, staining was significantly more intense on GBM cells allowing discrimination between both types of cells as demonstrated by the images presented in Fig. 3.

**Fig. 4:**
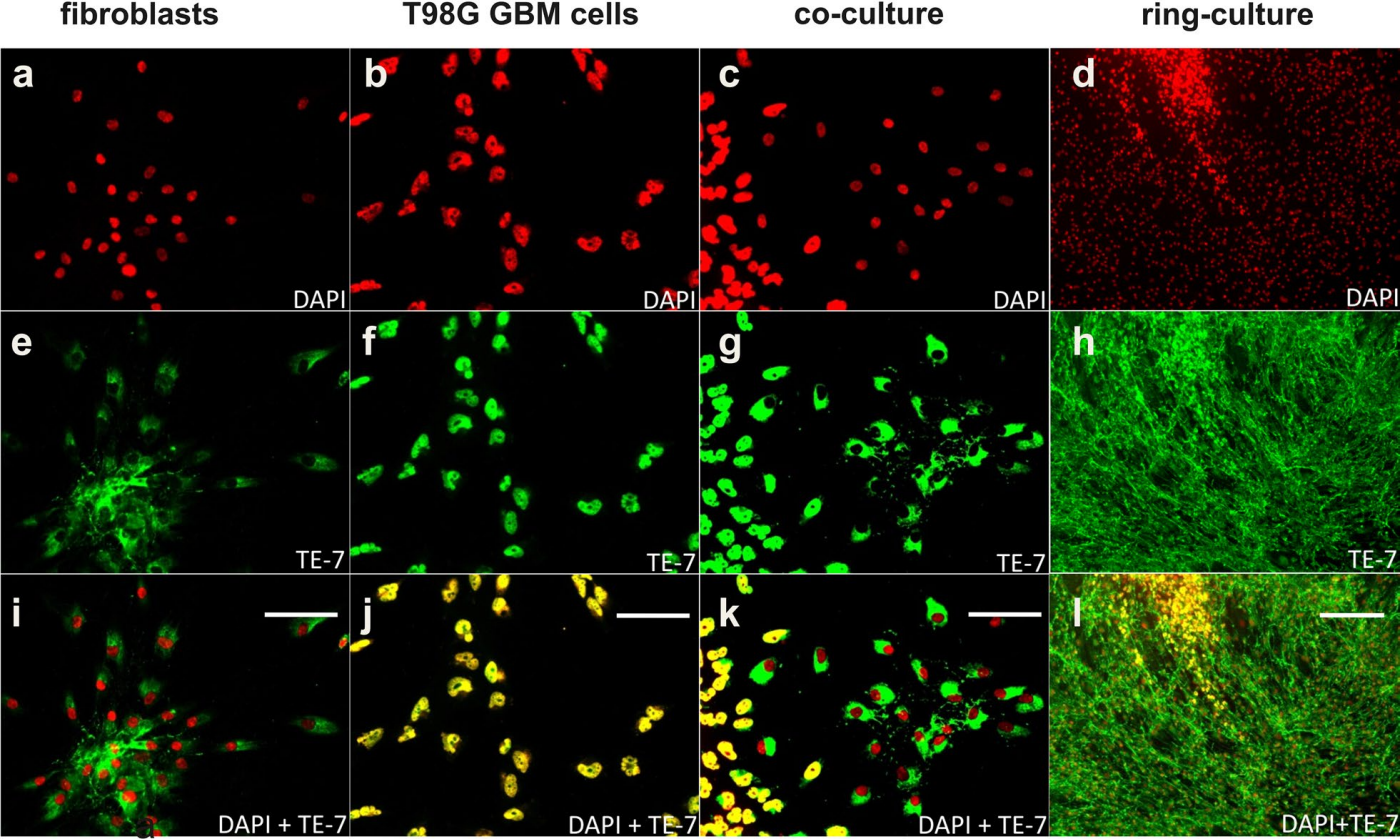
TE-7 staining of T98G cells and fibroblasts in mono-, co- and in ring co-culture. The figure shows staining in mono cultures of fibroblasts (P0375) with DAPI (**a**), with a TE-7-specific antibody (**e**) and the merged image (**i**) and the corresponding images of staining of T98G cells (**b**, **f**, **j**). Images **c**, **g** and **k** were taken from a co-culture of both cells and **d**, **h** and **f** shows an image of a ring culture at low magnification. Fibroblasts appear with green staining in the cytoplasm and red nuclei, whereas T98G cells are presented by their yellow stained nuclei in the merged images. Scale bars **a**–**c**, **e**–**g**, **i**–**k** 30 µm, **d**, **h**, **i** 120 µm

Unfortunately, discrimination between fibroblasts and cells from the glioblastoma cell line T98G was not possible using the nestin-specific antibody. We found a solution, using an anti-body directed against TE-7 which stains an unknown antigen specific for fibroblasts [18]. As demonstrated in Fig. 4 the TE-7 specific anti-body did also stain the nuclei of T98G cells, but did not detect antigen in their cytoplasm. Therefore, counterstaining with DAPI (color-coded in red) and TE-7 (color-coded in green) results in a yellow color of T98G nuclei and fibroblasts appear in green (cytosol) with red nuclei. At this point it should also be noted that the TE-7 specific antibody did also stain cells in primary GBM cultures which is the reason why this antibody had not been used for co-cultures with primary cells.

### Colony formation of T98G tumor cells in ring culture with fibroblasts under the influence of carnosine

**Fig. 5:**
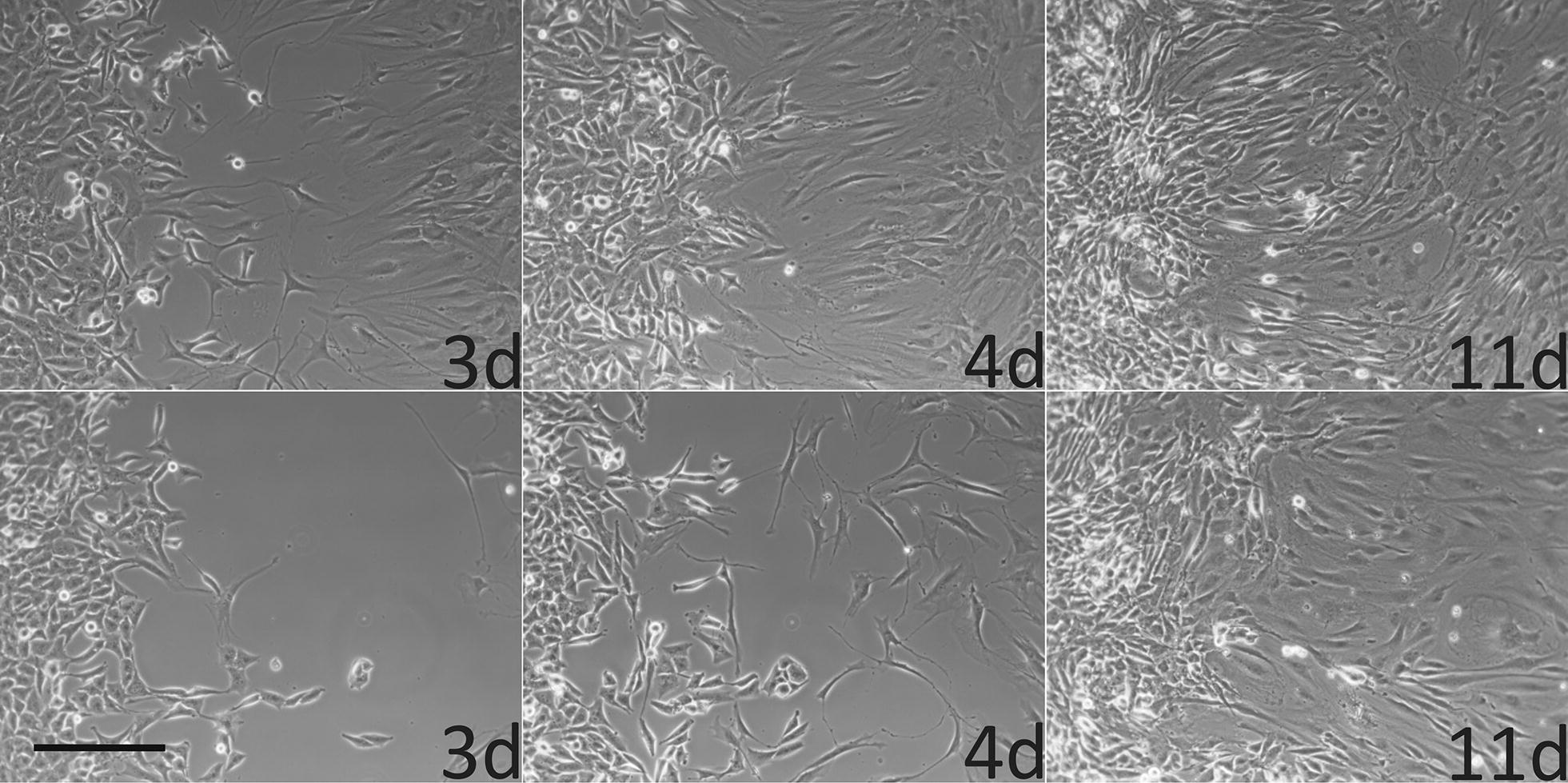
Cells migrating into the gap after removal of the cloning ring in a co-culture of T98G cells and fibroblasts. T98G cells (2500) were placed inside the cloning ring and fibroblasts (P0375; 50.000) outside of it. Migration of cells into the gap left after removal of the cloning ring 3, 4 and 11 days after ring removal with fibroblasts on the right side and T98G cells to the left as detected by bright field microscopy is presented. In the upper panel cultures were incubated in medium without carnosine and in the lower panel in medium containing 50 mM carnosine. Scale bar: 30 µm

**Fig. 6:**
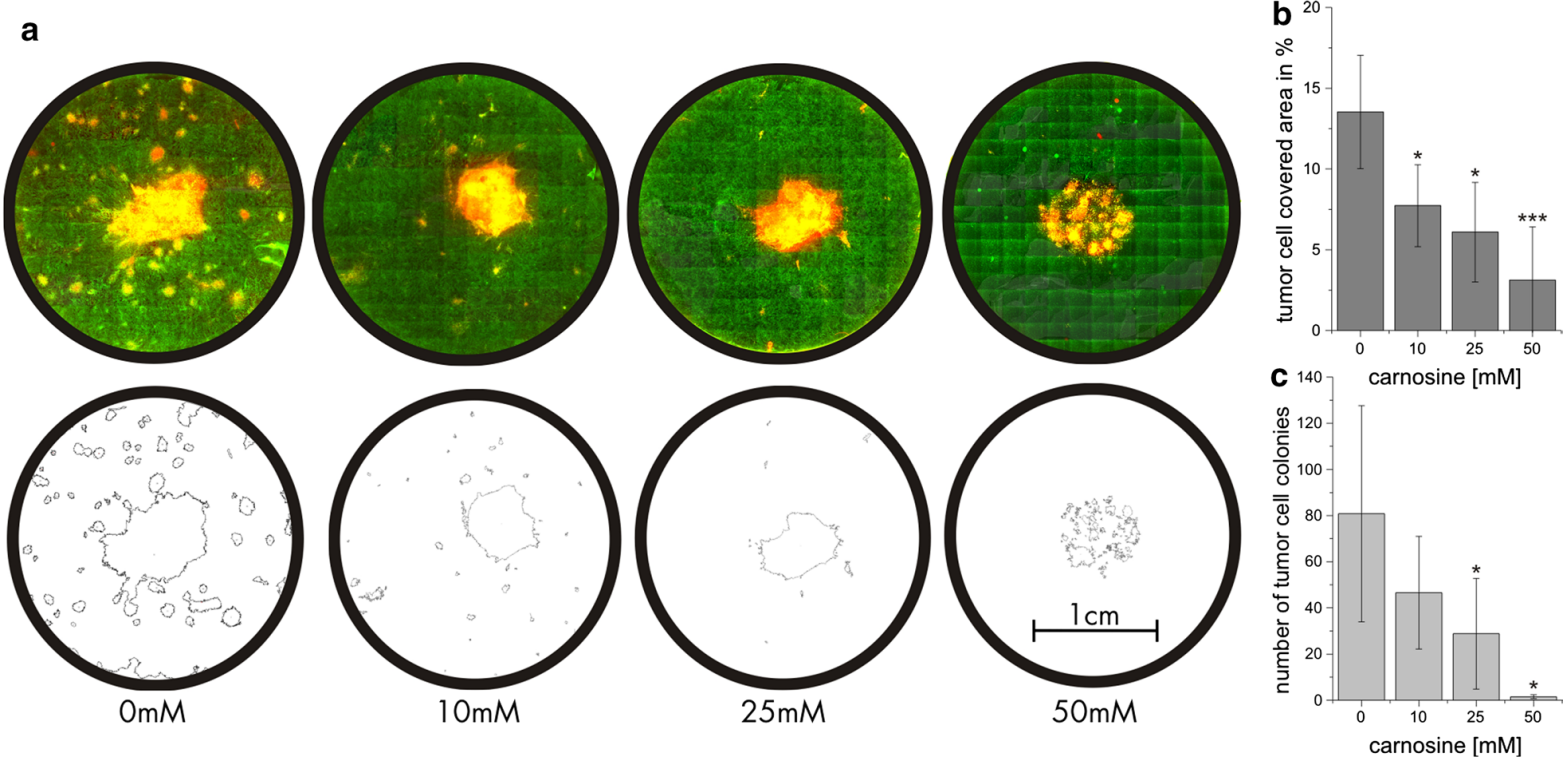
Colony formation of T98G cells in a fibroblast layer in the presence of different concentrations of carnosine. T98G cells (2500) were placed inside the cloning ring and fibroblasts (P0375; 50.000) outside of it. The ring was removed 4 h later and after 4 weeks of cultivation in the presence of different concentrations of carnosine the cultures were fixated and stained with DAPI and a TE-7 specific antibody. Fluorescence images are presented in the upper part of panel **a** and pictures generated by ImageJ for the determination of area occupancy and the number of colonies formed is presented in the lower part. On the right side of the graph, area occupancy (**b** 100% equals the whole well area) and the number of tumor cell colonies formed (**c**) is presented. Statistical significance using data obtained from 8 to 10 independently treated cultures for each concentration of carnosine was determined using Student’s t-test with: *p < 0.05; ***p < 0.0005

Next we asked whether T98G cells seeded inside a cloning ring can migrate into a surrounding layer of fibroblasts and whether migration and development of tumor cell colonies inside the fibroblast layer is influenced by carnosine. Therefore, ring co-cultures were established with T98G cells inside the cloning ring and fibroblasts (P0375) outside of it. After removal of the ring, cells first have to migrate towards each other filling the gap left after ring removal. As presented in Fig. 5 cells are migrating to fill the gap within 4–11 days. As can be seen, both, fibroblasts (right side) and T98G cells are able to migrate towards each other, filling the gap between day 4 and day 11 in the absence of carnosine. In the presence of 50 mM carnosine migration is impaired in both types of cells as shown in the image taken after 4 days. At day 11 the gap gets filled, too, but it seems that mainly fibroblasts are located in the previous gap (Note: This experiment resembles a classical scratch assay). In a following series of experiments ring co-cultures of T98G cells and fibroblasts (P0375) incubated for 4 weeks in different concentrations of carnosine (0, 10, 25 and 50 mM) were fixated and stained with DAPI and the TE-7 specific antibody. After fluorescence microscopy the number of colonies formed by T98G cells and the area occupied by tumor cells was analyses using ImageJ. In Fig. 6a example fluorescence images of cultures and their corresponding ImageJ derived images are presented as well as the result of the determination of area occupancy (Fig. 6b) and the measurement of the number of colonies formed (Fig. 6c). As can be seen, the size of the area occupied by tumor cells and the number of colonies are decreasing with increasing concentrations of carnosine. In the absence of carnosine tumor cells occupied 13.5% ± 3.5% of the plate. Already in the presence of 10 mM carnosine area occupancy was significantly decreased to 7.7% ± 2.5% (p < 0.05) and diminished further by increasing concentrations of carnosine (25 mM: 6.0% ± 3.0%, p < 0.05; 50 mM: 3.1% ± 3.3%, p < 0.0005). In addition, the number of colonies decreased with increasing concentrations from 80.8 ± 46.8 colonies in the absence of carnosine to 46.6 ± 24.4 colonies (10 mM; not significant), 28.8 ± 24.0 (25 mM, p < 0.05) and to 1.5 ± 0.8 colonies (50 mM, p < 0.05). This clearly demonstrates that carnosine inhibits the potential of T98G cells to infiltrate the fibroblast layer.

### Colony formation of primary glioblastoma cells in ring culture with fibroblasts under the influence of carnosine

In order to verify that the effect of carnosine is not restricted to a cell line but can also be seen with tumor cells derived from a patient, we also performed ring co-culture experiments with two patient-derived tumor cell cultures (P0383 and P0431) and fibroblasts from the same patient (P0385 and P0433, respectively). Again, tumor cells were seeded inside the cloning ring and fibroblasts outside of it. After 4 weeks of cultivation cells were stained with DAPI and a nestin-specific antibody and analyzed using ImageJ. Surprisingly, we did not detect colony formation inside the fibroblast layer even in the absence of carnosine. On the other hand, the tumor occupied area was significantly reduced already at a concentration of 10 mM carnosine. As the two primary cultured glioblastoma cells occupied different areas in the absence of carnosine, we set the area occupied in the absence of carnosine for each cell culture as 100% in order to compare the effect of carnosine and to determine an average. We found a reduction of the occupied area to 68.1% ± 11.3% (p < 0.05) at a concentration of 10 mM carnosine, to 70.6% ± 3.1% (p < 0.0005) at 25 mM and to 18.7% ± 3.0% (p < 0.0005) at 50 mM. Comparable to the experiments with T98G cells and fibroblasts presented in Fig. 6, the space previously occupied by tumor cells becomes occupied by the fibroblasts. (For comparison: setting the occupied area to 100% in the experiment with T98G cells the reduction is 57.0% ± 18.5% at 10 mM, 44.4% ± 22.2% at 25 mM and 23.0% ± 24.4% at 50 mM carnosine).

## Discussion

As outlined in the introduction, we and others have shown that the naturally occurring dipeptide carnosine inhibits the growth of cancer cells in vitro and in vivo [14], whereas beneficial effects have been observed in cultured human fibroblasts [19]. Using a colorimetric assay (tetrazolium compound [3-(4,5-dimethylthiazol-2-yl)-5-(3-carboxymethoxyphenyl)-2-(4-sulfophenyl)-2H-tetrazolium, inner salt; MTS]) and human dermal fibroblasts, Ansurudeen et al. also demonstrated a greater number of viable cells in the presence of 50 mM carnosine after 24 h incubation [20]. Unfortunately, it is not traceable whether the fibroblasts used by Ansurudeen et al. were from juvenile foreskin or from adult skin. In addition, the experiments performed by Holliday and McFarland were done by using a fibroblast cell line established from human foreskin of a newborn male (HFF-1) and a second cell line established from lung tissue of a male at 14 weeks gestation (MRC-5). Considering using carnosine for the treatment of elderly patients the question had to be answered whether fibroblasts isolated from adults or even senescent patients may behave different to fibroblasts isolated from fetal or newborn human tissue. In our experiments presented in Fig. 1 we did not see a measurable beneficial effect of carnosine on fibroblast viability, although it is interesting to note, that fibroblasts cultivated in 50 mM carnosine appeared to be rejuvenated compared to fibroblasts cultivated in the absence of carnosine in accordance to the observations of McFarland and Holiday [21]. We also did not see any correlation between age of the patient, the tissue of origin or gender, though it has to be realized that the number of samples may be too low for such an analysis and most of our patients have been of comparable age. More importantly, a prolonged cultivation of fibroblasts as in the co-culture experiments demonstrates that the fibroblasts are alive and able to occupy the space left by dying glioblastoma cells even under the highest concentration of carnosine employed (50 mM). It is also very interesting to note that we observed complete cell death of T98G tumor cells in long term culture incubating the cells at a concentration of 75 mM carnosine (Fig. 2). In previous experiments, cells were usually kept in the presence of carnosine for 24, 48 or 96 h [13] but not for 2 weeks. This is interesting, as shorter exposure times in previous experiments resulted in reduced proliferation but not complete elimination of tumor cells. Which processes are responsible for reduced tumor cell proliferation are under the influence of carnosine are still unknown [14]. However, it is an interesting question whether the processes which reduce proliferation after short term exposure may finally lead to cell death when the tumor cells are exposed to carnosine for a longer period of time.

In order to discriminate tumor cells from fibroblasts in ring co-culture experiments several markers were tested including glial fibrillary acidic protein (GFAP) which did also stained fibroblasts in accordance with observations made by others [22]. We finally identified that nestin-staining was suitable to discriminate primary cultured tumor cells from patient-derived fibroblasts. Nestin is a class VI intermediate filament protein and a marker for neural stem cells. In addition, it has been reported as a cancer stem cell-specific marker [23] and a recent meta-analysis performed by Lv et al. [24] demonstrated that increased expression of nestin is positively associated with higher histological grade in glioma patients. This analysis also indicated that patients with higher nestin expression are prone to recurrence and glioma cell infiltration into intact brain tissue. Surprisingly, in our ring co-culture experiments T98G cells, which did not express nestin, as previously reported by other investigators [25, 26], gave rise to many colonies in the surrounding fibroblast layer which was not the case when primary cultures with a high expression of nestin were cultivated with fibroblasts. Although speculative, one interpretation could be that the primary cultures we used were of very early passages (Passage 1 and 5) and not as rapidly growing as T98G cells which is also reflected by their higher resistance towards carnosine (Fig. 1) as carnosine exerts its action mainly on metabolic highly active tumor cells [27]. Nonetheless, the results presented in Fig. 6 clearly demonstrate that colony formation is significantly inhibited when invasively grown glioblastoma cells are treated with carnosine. In the last years, a so-called “go or grow concept” has been discussed, assuming that proliferation and migration are mutually exclusive phenomena in cancer cells [4]. Studies confirming this hypothesis are for example observations made in breast cancer cell lines in which overexpression of Homeobox Protein C9 (HOXC9) resulted in increased invasiveness but at the same time inhibited proliferation [28]. Another example is the observation that enforced expression of Y-box binding protein-1 (YB-1) in non-invasive breast epithelial cells induces an epithelial–mesenchymal transition (EMT) resulting in an enhanced metastatic potential but at the same time reduces proliferation [29]. Unfortunately, the exact mechanisms and down-stream targets responsible for either proliferation or invasion mediated by HOXC9 or YB-1 signaling are still unknown. More importantly, our results demonstrate that carnosine does not inversely influence proliferation and invasion. Up to now, the mechanisms responsible for the dipeptides anti-proliferative effect, which has been confirmed in several studies with different types of cancer cells [11–13, 30] are still not understood. With regard to carnosine’s effect on migration and invasion it has been discussed that it involves regulation of matrix metalloproteinases (MMPs) [15], but more experiments are certainly needed to properly address this question.

## Conclusions

This study demonstrates that carnosine’s anti-proliferative effect is not accompanied by increased invasion as suggested by the so-called “go-or-grow” concept. In fact, the dipeptide can inhibit tumor cell migration, which is especially important for the treatment of highly infiltrating and metastasizing tumors such as IDH-wildtype glioblastoma. In addition, the co-culture model presented is a valuable alternative to the commonly used scratch or Boyden chamber assays.

## Additional files

**Additional file 1: Table S1.** Patients and patient derived cell cultures.

**Additional file 2.** Setup of ring-cultures.

## Abbreviations

CTB: CellTiter-Blue Cell Viability Assay
CTG: CellTiter-Glo Luminescent Cell Viability Assay
GBM: IDH1R132H-non-mutant glioblastoma
GFAP: glial fibrillary acidic protein
HOXC9: Homeobox Protein C9
IDH: isocitrate dehydrogenase
MGMT: *O*-6-methylguanine-DNA methyltransferase
MTS: 3-(4,5-dimethylthiazol-2-yl)-5-(3-carboxymethoxyphenyl)-2-(4-sulfophenyl)-2H-tetrazolium, inner salt
YB-1: Y-box binding protein-1
WHO: World Health Organization
