# Supplementary material for: Carnosine selectively inhibits migration of IDH-wildtype glioblastoma cells in a co-culture model with fibroblasts
Source: Cancer Cell Int. 2018 Aug 13;18:111. doi: 10.1186/s12935-018-0611-2 (PMC6090706; doi:10.1186/s12935-018-0611-2)
Supplement: Supplementary file 1 — Additional file 1: Table S1. Patients and patient derived cell cultures. [file 12935_2018_611_MOESM1_ESM.docx]

**Table S1 Patients and patient derived cell cultures**

| patient | cell type | Label | sex and age | MGMT status GBM | IDH status GBM |
| --- | --- | --- | --- | --- | --- |
| 1 | Fibr. (P) | P0375 | m, 66 | - | - |
| 2 | GBM | P0383 | f, 76 | mildly positive | negative |
|  | Fibr. (P) | P0384 |  |  |  |
|  | Fibr. (G) | P0385 |  |  |  |
| 3 | Fibr. (G) | P0408 | f, 41 | positive | negative |
|  | GBM | P0410 |  |  |  |
| 4 | Fibr. (P) | P0425 | f, 67 | mildly positive | negative |
|  | GBM | P0424 |  |  |  |
| 5 | GBM | P0411 | m, 68 | positive | negative |
|  | Fibr. (P) | P0412 |  |  |  |
| 6 | GBM | P0431 | m, 66 | positive | negative |
|  | Fibr. (G) | P0433 |  |  |  |
| 7 | GBM | P0441 | f, 59 | negative | negative |
|  | Fibr. (G) | P0443 |  |  |  |
| 8 | GBM | P0446 | m, 65 | positive | negative |
|  | Fibr. (G) | P0447 |  |  |  |
| 9 | GBM | P0451 | f, 74 | positive | negative |
|  | Fibr. (G) | P0452 |  |  |  |
| 10 | GBM | P0454 | m, 70 | negative | negative |
|  | Fibr. (G) | P0455 |  |  |  |

Primary cultures of glioblastoma cells (GBM) and fibroblast cultures either isolated from periost (Fibr. (P)) or from galea (Fibr. (G)) are shown together with patients’ age and sex and the status of promoter methylation of MGMT and whether the tumors are positive or negative for IDH mutation R132H as determined by pathology.
