# Supplementary figures and images for: Carnosine selectively inhibits migration of IDH-wildtype glioblastoma cells in a co-culture model with fibroblasts
Source: Cancer Cell Int. 2018 Aug 13;18:111. doi: 10.1186/s12935-018-0611-2 (PMC6090706; doi:10.1186/s12935-018-0611-2)

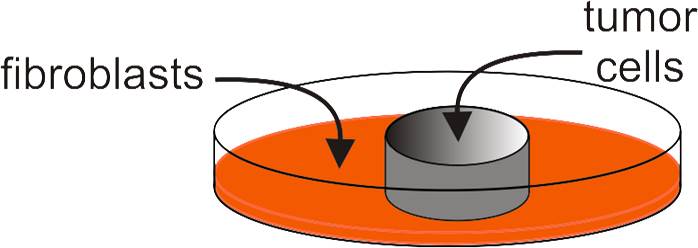

Supplement: Supplementary file 2 — Additional file 2. Setup of ring-cultures. [file 12935_2018_611_MOESM2_ESM.jpg]
